# Case Report: Genetic profiling of small intestine metastasis from poorly differentiated non-small cell lung cancer: report of 2 cases and literature review of the past 5 years

**DOI:** 10.3389/fonc.2023.1265749

**Published:** 2023-11-21

**Authors:** Mengqin Wang, Gang Chen, Jiang Luo, Zhipeng Fan, Yuying Liu, Conghua Xie, Yan Gong

**Affiliations:** ^1^ Department of Radiation and Medical Oncology, Zhongnan Hospital of Wuhan University, Wuhan, China; ^2^ Hubei Key Laboratory of Tumor Biological Behaviors, Zhongnan Hospital of Wuhan University, Wuhan, China; ^3^ Tumor Precision Diagnosis and Treatment Technology and Translational Medicine, Hubei Engineering Research Center, Zhongnan Hospital of Wuhan University, Wuhan, China

**Keywords:** poorly differentiated NSCLC, small intestine metastasis, genetic profiling, radiotherapy, gene mutation

## Abstract

**Background:**

Poorly differentiated non-small cell lung cancer (NSCLC) is characteristic of high rate of distant metastasis and late stages at diagnosis. Small intestine metastasis is a rare but severe complication of lung cancer with a high rate of mortality. However, there is currently a lack of genetic profile studies on the small intestine metastasis of lung cancer.

**Case presentations:**

We present 2 cases of male patients in their 60s with primary NSCLC of low differentiation, initially with no distant metastasis detected. Biopsy samples were obtained from the primary pulmonary lesions, and both patients received systematic radiotherapy (RT) and chemotherapy. However, both cases presented with abdominal pain and distension, and immunohistochemistry of small intestine biopsy samples obtained by endoscopy confirmed lung cancer metastasis. Next generation sequencing was used to explore the genetic profiles from the biopsy samples of both the primary pulmonary lesions and small intestine metastases. The correlated genes responsible for the small intestine metastasis from poorly differentiated NSCLC in these 2 patients included TP53, LRP1B, and FGFR2. The reports of small intestine metastasis from poorly differentiated NSCLC with the past 5 years were systematically reviewed and summarized subsequently.

**Conclusions:**

Poorly differentiated NSCLC with small intestine metastases, while rare, substantially impacts the prognosis and poses major challenges for diagnosis and treatment. Through comparisons of genetic profiles between patients and in the same patient before and after metastasis, we identified the mutations in genes such as TP53, LRP1B, and FGFR2, which were correlated with the occurrence and progression of poorly differentiated NSCLC, as well as its small intestinal metastasis. This discovery has the potential to guide clinicians in developing personalized treatment plans through the manipulation of targeted and radiation therapies.

## 1 Introduction

Global cancer statistics revealed that lung cancer is the leading cause of cancer-related mortality worldwide (1). Non-small cell lung cancer (NSCLC) accounts for the majority (approximately 85%) of lung cancer cases. Compared with well differentiated ones, poorly differentiated NSCLC had an increased mortality by 83% and a 2.1-fold increase of recurrence due to its rapid growth rate and high metastasis propensity (2). More than 60% poorly differentiated NSCLC patients suffer from distant metastasis (3), including lymph nodes, liver, adrenal glands, bone, and brain. The occurrence of gastrointestinal tract metastasis is relatively uncommon, with only 1.77% reported, but it severely impacts the survival of lung cancer patients (4). A retrospective analysis of 366 lung cancer patients with gastrointestinal metastasis revealed that over half of them passed away within 3 months from diagnosis, with a median overall survival of 2.8 months (5). Among them, small intestine metastasis was further rare, reported in 11.9% of gastrointestinal cases, but substantially shortened the survival of lung cancer patients (6).

With technological development, next generation sequencing (NGS) is increasingly applied to detect genetic alterations and guide personalized treatments for patients in clinic. It has been demonstrated that mutations in driver genes such as EGFR, ALK, and KRAS are associated with metastases in various sites in NSCLC, including the brain, bone, liver, and lungs (7), but not the intestine. Reports and studies on lung cancer with small intestine metastasis remain relatively limited. Early detection or prediction is critical and necessary to improve efficacy and patient survival.

In this report, we presented 2 cases of poorly differentiated NSCLC with small intestine metastasis. By juxtaposing their NGS results, we unearthed the most correlated genes, including but not limited to, TP53, LRP1B, and FGFR2. Investigating genetic profiles in NSCLC with small intestine metastasis is crucial for early detection and prevention, and may provide insights to the underlying molecular mechanisms and potential therapeutic targets.

## 2 Case presentations

### 2.1 Case 1

The patient, a 61-year-old male, presented to the local hospital in April 2022 with complaints of dizziness and fatigue. A left upper lobe partial resection with lymph node clearance was performed after a 21 mm x 15 mm mass was found on a chest computed tomography (CT). Postoperative pathology revealed infiltrating adenocarcinoma with low to moderate differentiation. Immunohistochemistry revealed partial positive staining for CK7, focal positive staining for NapsinA, and TTF-1, negative staining for CK20. After thorough examination, the diagnosis of the malignant tumor in the left lung was confirmed as pT1cN0M0. Four cycles of PP regimen (Cisplatin + Pemetrexed) chemotherapy were given postoperatively, followed by radiotherapy (RT) of 56 GY/25 F from September 10 to November 14, 2022.

**Figure 1:**
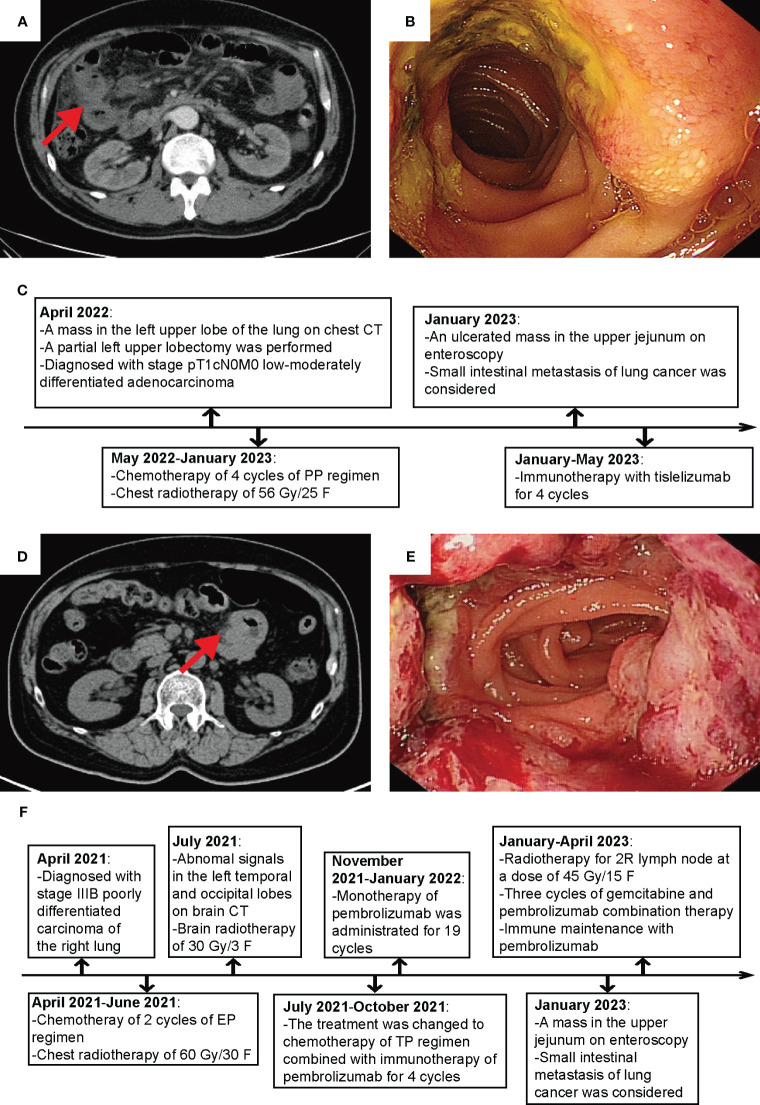
Clinical and imaging features of the 2 cases. **(A)** Abdominal CT image of Patient 1. **(B)** Small intestine endoscopy image of Patient 1. **(C)** Flowchart of Patient 1’s diagnosis and treatment course. **(D)** Abdominal CT image of Patient 2. **(E)** Small bowel endoscopy image of Patient 2. **(F)** Flowchart of Patient 2’s diagnosis and treatment course.

On January 6, 2023, the patient was admitted to our hospital due to abdominal pain and distension. CT examination of the abdomen and pelvis showed segmental thickening of the small intestinal wall in the right upper abdomen and multiple enlarged lymph nodes in the pelvic and inguinal regions (**Figure 1A**). On January 16, 2023, endoscopic examination of the small intestine revealed an ulcerated tumor in the jejunum, occupying approximately 2/3 of the lumen, located approximately 100 cm from the pylorus (**Figure 1B**). Histopathological examination of the biopsy specimen showed a high-grade cancer-like lesion in the submucosal layer of the small intestine. Immunohistochemistry demonstrated weakly positive expression of CK, positive expression of CK7 and Vimentin, negative expression of NapsinA, TTF-1, CK20. The diagnosis of undifferentiated cancer was considered.

From January 20 to May 19, 2023, the patient underwent 6 cycles of maintenance therapy with Tislelizumab. The therapeutic response was assessed every 2 cycles and classified as stable disease (SD) (**Figure 1C**).

### 2.2 Case 2

The patient, a 65-year-old man, was admitted to the hospital on April 6, 2021 due to a one-month history of coughing and phlegm. Chest CT scans revealed soft tissue nodules in the upper and middle lobes of the right lung, as well as soft tissue masses in the right pulmonary hilum and multiple enlarged lymph nodes in the mediastinum and right pulmonary hilum. No other malignant tumors or signs of metastasis were found in other parts of the body on PET/CT. A sample of the right lung mass and the 7th groups of lymph nodes were taken for pathological examination, which revealed poorly differentiated cancer. Immunohistochemistry showed positive expression of CK7, along with negative expression of TTF-1. The final diagnosis was low-grade lung cancer in the right lung, staging as T4N2MO. The patient had a smoking history of more than 40 years, averagely smoking 20 cigarettes per day.

From April 29 to June 18, 2021, the patient received 2 cycles chemotherapy of EP regimen (Etoposide + Nedaplatin) and chest RT with a total dose of 60 GY/30 F. On July 21, 2021, a review of the enhanced CT scan of the brain showed abnormal signals in the left temporal and parietal lobes, suggesting metastasis. Therefore, the patient received brain RT with a total dose of 30 GY/3 F, and the treatment plan was changed to chemotherapy of TP regimen (Albumin paclitaxel + Nedaplatin) combined with immunotherapy of Pembrolizumab for 4 cycles. The efficacy evaluation showed SD in the lungs and complete response (CR) in the brain. Starting from November 17, 2021, the patient received single-agent Pembrolizumab as maintenance for 19 cycles, and the efficacy evaluation showed SD.

On January 4, 2023, the patient returned for a abdominal CT due to hoarseness and abdominal distension, which showed multiple enlarged lymph nodes in the 2R region of the mediastinum, thickening of the wall of the jejunum in the upper left abdomen (**Figure 1D**). On January 13, 2023, a small intestine endoscopy revealed a luminal growth in the upper part of the jejunum, located approximately 35 cm from the pylorus (**Figure 1E**), and biopsy confirmed poorly differentiated cancer. Immunohistochemistry showed positive staining for CK7 and NapsinA, negative staining for TTF-1 and CK20. The possibility of poorly differentiated lung cancer with jejunal metastasis was considered.

From January 12, 2023, the patient received RT for swollen mediastinal lymph nodes in 2R region, administering a dose of 45 GY/15 F. Between February 8 and March 25, 2023, the patient underwent chemotherapy with Gemcitabine in combination with immunotherapy of Pembrolizumab for 3 cycles. Upon evaluation in April 2023, the therapeutic effect was classified as SD, which prompted a treatment regimen of immunotherapy maintenance with Pembrolizumab. The patient is currently receiving regular treatment (**Figure 1F**).

### 2.3 Genetic profiling of the 2 patients

**Figure 2:**
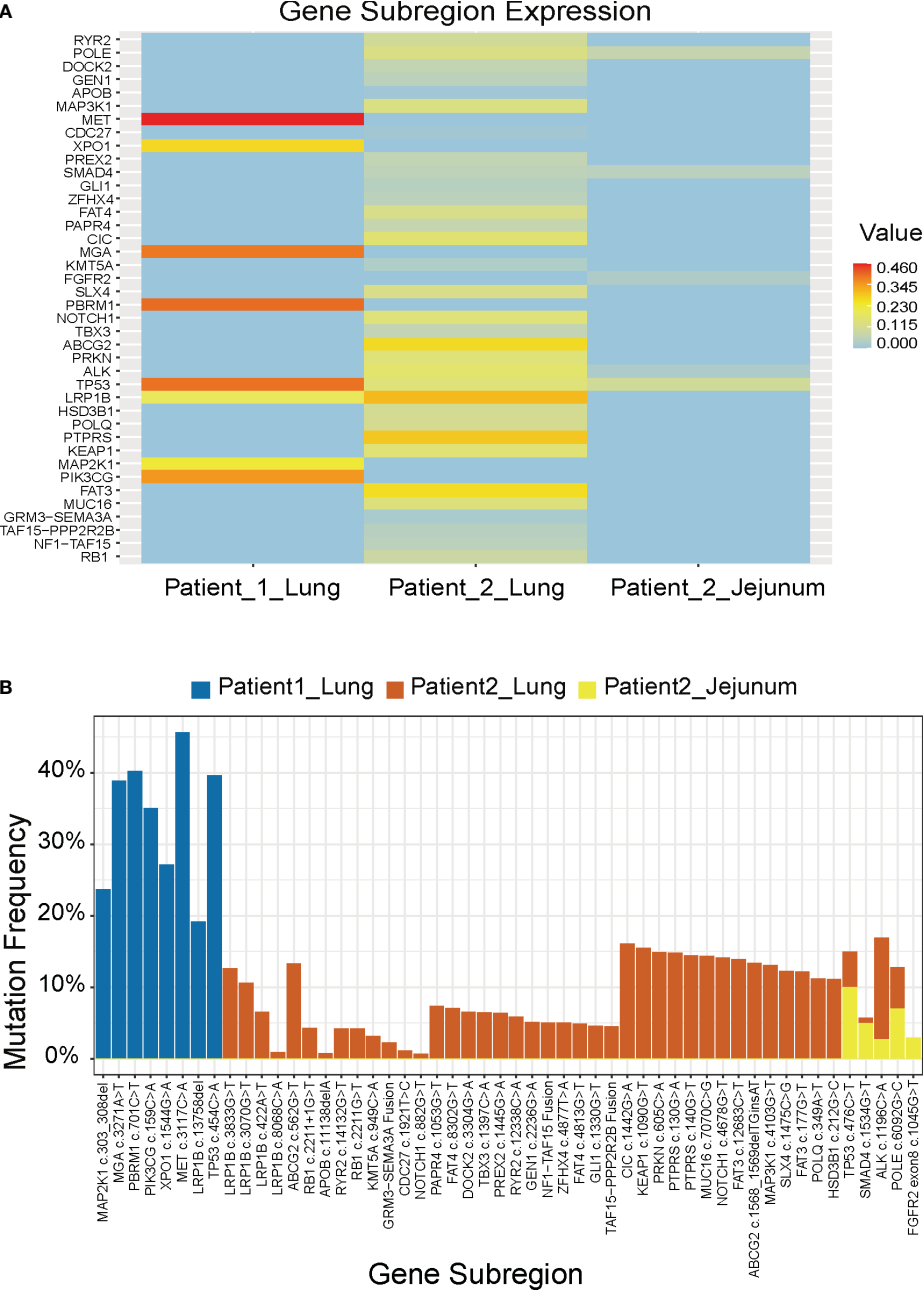
Molecular features of the 2 patients. **(A)** Mutation genes and their mutation frequencies. **(B)** Comparison of mutation sites and their mutation frequencies.

We compiled the NGS results of both patients, including the lung tumor of patient 1 and the lung and small intestine masses of patient 2 (**Figure 2A**). In Patient 1, the top 5 genes with the highest mutation frequency were TP53, MGA, PBRM1, PIK3CG, and MET, whereas in Patient 2, they were TP53, ABCG2, LRP1B, FAT3, and PTPRS. Furthermore, we observed that both cases exhibited some shared genetic mutations, such as TP53 and LRP1B, while Patient 2 experienced a novel gene mutation, FGFR2, after small intestine metastasis (**Figure 2B**).

## 3 Discussion and literature review

**Figure 3:**
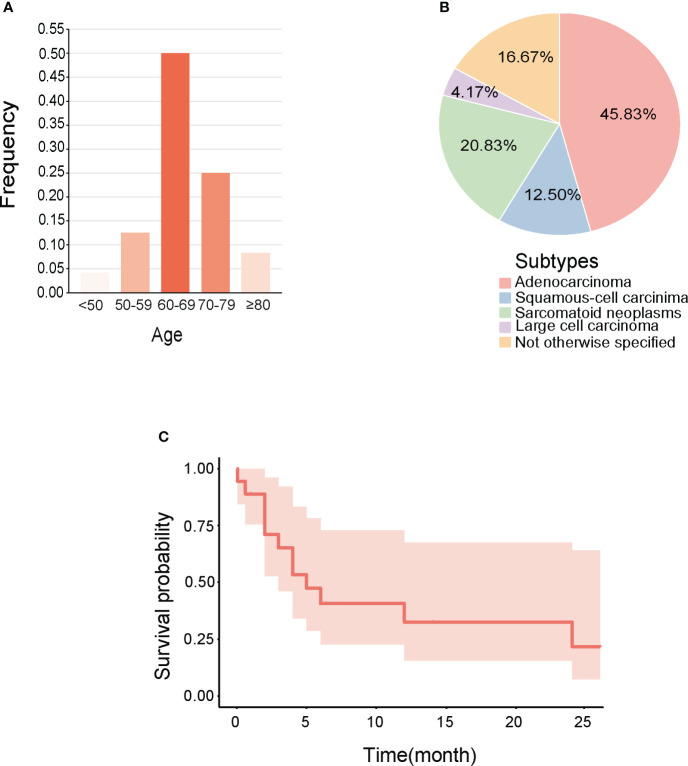
Clinical features of small Intestine metastasis from NSCLC reported in recent 5 years. **(A)** Age range. **(B)** Pathological types. **(C)** Survival curves made for integrating the prognosis.

Our analysis of reported cases small intestine metastasis from NSCLC over the past 5 years indicated that most patients were over 60 years old (**Figure 3A**), the primary pathological type was mostly adenocarcinoma (**Figure 3B**), and that the initial symptoms of metastatic disease typically manifested as abdominal pain and anemia, with a poor prognosis that often resulted in death within 6 months (**Figure 3C**, **Table 1**).

**Table 1:** Reported cases of small intestine metastasis from primary NSCLC within the past 5 years.

Age Gender	Type of histological	Symptoms	Other metastasis	Treatment	Prognosis	Author
62 M	Adenocarcinoma	Abdominal pain, anemia	Lymph node	Chemotherapy	Died 3 months after metastasis	Ogasawara et al. (2022) (8)
86 M	Non-small cell cancer	Systemic syndrome, anemia	–	Palliative treatment	Died after 4 months	C. Saldaña-Dueñas et al. (2019) (9)
68 M	Adenocarcinoma	Anemia	Lymph node, liver	Chemotherapy	Survived at 5th month	C. Saldaña-Dueñas et al. (2019) (9)
59 F	Adenocarcinoma	Bloody stools, anaemia	Adrenal gland	Small intestine resection, immunotherapy	–	D. Kosciuszek et al. (2022) (10)
61 M	Squamous-cell carcinoma	Abdominal distention, weight loss	Stomach	Surgery, chemotherapy	Died 6 months after surgery	Li et al. (2018) (11)
60 M	Adenocarcinoma	Edema	Lymph nodes, adrenal glands, chest wall, bone	Surgery	Died at 19th day following diagnosis	Wang et al. (2018) (12)
64 M	Adenocarcinoma	Melena, weight loss, anaemia	Lymph node, liver	Palliative care	–	Kosasih et al. (2019) (13)
73 F	Adenocarcinoma	Weakness, melena, anaemia	Lymph node, liver	Chemo-radiotherapy	–	Kosasih et al. (2019) (13)
81 F	Poorly differentiated neoplasm	Abdominal pain, anaemia	Liver, sigmoid colon	Surgery	Death on the second day of surgery	P. Misiakos et al. (2019) (14)
62 M	Adenocarcinoma	Abdominal pain	Brain, bone, liver	Targeted treatment	Died 2 months later after metastasis	Zhu et al. (2021) (15)
70 M	Non-small cell cancer	Abdominal pain	Brain, liver	Surgery, chemotherapy	Aive after 1 month of follow-up	Sautto et al. (2022) (16)
41 M	Large cell carcinoma	Abdominal pain, vomiting	Lymph nodes, pleura, adrenal glands, bone	Surgery	–	Kim et al. (2020) (17)
65 M	Squamous cell carcinoma	Abdominal pain	Stomach, rectum	Surgical resection	Alive after 2 years of follow-up	Zhu et al. (2022) (18)
70 M	Pleomorphic carcinoma	Fecal occult blood, anaemia	Stomach, peritoneum	Surgery, immunotherapy	Cancer-free state at 26th month	Suzuki et al. (2022) (19)
61 M	Epidermoid carcinoma	Abdominal pain, vomiting, constipation	–	Surgery	Died 1 year later after progress	Rejab et al. (2022) (20)
69 M	Adenocarcinoma	Bloating, decreased flatus	–	Targeted treatment	Alive at 14th month of follow-up	Chen et al. (2018) (21)
75 M	Adenocarcinoma	Melena, anemia	Liver, bone, brain	Palliative care	Died 2 months later after diagnosis	Ohira et al. (2019) (22)
65 F	Adenocarcinoma	Intestinal obstruction	–	Surgery	Relapse after 2 years	Pierro et al. (2019) (23)
77 M	Adenocarcinoma	Poor oral intake, nausea, vomiting, diarrhea	–	Surgery	Died for postoperative complications	Nguyen et al. (2019) (24)
71 M	Undifferentiated pleomorphic sarcoma	Fever, upper abdominal pain	Brain	Surgery	Alive at 10th month of follow-up	Eto et al. (2021) (25)
55 M	Carcinoma with sarcomatoid differentiation	Epigastric pain, melena	Lymph nodes, adrenal gland	Surgery	Died 4 months after surgery	Xie et al. (2020) (26)
61 M	Sarcomatoid carcinoma	Melena, fever, anaemia	–	–	Died 5 months after diagnosis	Xie et al. (2020) (26)
66 M	Sarcomatoid carcinoma	Melena, anaemia	Epiglottis, lymph nodes, adrenal gland	Immunotherapy, targeted treatment	–	Gao et al. (2022) (27)
57 M	Undifferentiated sarcoma	Acute abdomen	–	Surgery	Died 2 months after surgery	Pleština et al. (2019) (28)

We discovered that the mutations in these two patients were predominantly missense mutations. TP53 is the most commonly mutated gene in human cancer, and 70% of its mutations are missense in the DNA-binding domain (29). These mutations increase the incidence of distant metastasis, and are associated with reduced survival rates. TP53 serves as the major prognostic factor for early and advanced NSCLC, with an OS of 27 vs. 19 months (p < 0.001) (30). The co-occurring mutations with other genes such as EGFR, STK11, or KRAS have been shown to be independent biomarkers for immune checkpoint inhibitors. LRP1B was reported to reduce NSCLC cell proliferation and migration as a putative tumor suppressor, and its loss-of-function mutations might lead to oncogenesis (31). Compared with the NGS results before small intestine metastasis in patient 2, a newly emerging mutation of FGFR2 was found in the metastatic tumors. A retrospective analysis of 5,557 Chinese solid tumor patients showed that the frequency of FGFR2 alterations in lung cancer was 1%, and FGFR2 was highly expressed in NSCLC tissues, which was correlated with tumor metastasis and poor prognosis (32). Targeted therapy against FGFR2 has become a hot topic in lung cancer treatment, and the role of FGFR inhibitors including ponatinib, regorafenib, pazopanib, lenvatinib, and nintedanib in NSCLC is being intensively studied.

Between patients, there existed some distinct genetic subregions. The heterogeneity of either patient’s tumor might serve as one contributing factor, whereby diverse subclonal populations or mutational patterns might even coexist within a single patient’s sample. Tumor heterogeneity engenders high genetic, epigenetic, and phenotypic diversity among tumor cells, thus constituting a significant constraint on targeted therapies (33). In addition, tumor progression and evolution epitomize a dynamic process, wherein the course may vary among different patients, owing to disparities such as the tumor microenvironment. One aspect we can ascertain is that investigating shared mutated genes holds promise as a future avenue of researches.

Furthermore, the gene mutations in the primary lung tumors were essentially the same as those in the metastatic tumors. However, the mutation frequency decreased after the small intestine metastasis, leading us to speculate that the metastasis might cause selective pressure on the NSCLC cells, resulting in the survival and expansion of a specific subset of cells with lower mutation frequency. Only 20% of detected mutations were demonstrated to exhibit significant alterations following conventional therapy, including a tendency towards a decrease in mutation frequency.

NGS was not conducted on the small intestine metastasis in Patient 1 due to the inherently limited quantity of the endoscopically obtained tissue sample. The sample was utilized not only to ascertain the origin of the small intestine mass whether primary or metastatic, but also underwent additional immunohistochemical analyses. Subsequently, PD-L1 testing was performed to further establish immunotherapy indications. Determining the nature of the tumor and guiding immunotherapy decisions stand as paramount considerations for clinical practitioners. Furthermore, the incomparability of the patient’s blood sample results led us to abandon the notion of utilizing blood samples.

The treatment of intestine metastasis of lung cancer is not standardized in clinical practice. For patients with multiple metastases of lung cancer, RT and chemotherapy are preferred, and surgery can be an effective palliative treatment for severe complications such as bleeding, obstruction, and perforation. RT can reduce the risk of distant metastasis in early-stage NSCLC patients. In NSCLC cases with small intestine metastasis, RT can further control the growth of metastatic lesions and alleviate abdominal pain and digestive dysfunction. The efficacy of RT is influenced by genomic molecular variations that determine the radiosensitivity of cancer cells. Moreover, RT can enhance the efficacy of systemic treatments through its abscopal effects. In cases of metastatic lung cancer, concurrent local RT and metastatic targeting therapy can strengthen the sustained control of local metastases (34). Further research is needed to determine the optimal combination and sequence of RT and other treatments, such as chemotherapy or targeted therapy.

More molecular studies are required to elucidate the underlying mechanisms driving NSCLC migration to the small intestine. The mutated genes TP53 and LRP1B, shared by the 2 patients, along with the newly mutated gene FGFR2 that has emerged after small bowel metastasis, need to be further verified as the driving force behind the spread of lung cancer to the small intestine. Targeting the mutated genes or their downstream signaling pathways represents a breakthrough for exploring novel therapeutic strategies.

## 4 Conclusions

Metastasis is a complex multi-step process that involves various factors such as tumor microenvironment, genetic instability, and immune response. It is essential to perform molecular marker detection in NSCLC patients and clarify the correlation between the pattern of small intestine metastasis and driver gene mutations. This not only enables the identification of tumor progression, but also serves as a biological predictor of radiation sensitivity and an identifying factor for potential therapeutic targets, thereby guiding personalized treatment plans.

## 5 Patient’s perspective and informed consent

The emergence of small bowel metastasis signified disease progression for the patient. The patient exhibited high compliance during further examinations and treatments, and demonstrated an understanding of the importance of NGS testing. In the absence of therapeutic targets, the patient received immunotherapy. The efficacy assessments after every two cycles showed stable disease, with an improvement in the patient’s quality of life.

The patient in this case report had given consent for anonymous publication.

## Data availability statement

The datasets presented in this article are not readily available because of ethical/privacy restrictions. Requests to access the datasets should be directed to the corresponding authors.

## Ethics statement

The studies involving humans were approved by Zhongnan Hospital of Wuhan University. The studies were conducted in accordance with the local legislation and institutional requirements. The participants provided their written informed consent to participate in this study. Written informed consent was obtained from the individual(s) for the publication of any potentially identifiable images or data included in this article.

## Author contributions

YG: Project administration, Supervision, Writing – review & editing. MW: Visualization, Writing – original draft. GC: Data curation, Writing – original draft. JL: Investigation, Writing – original draft. ZF: Investigation, Writing – original draft. YL: Investigation, Writing – original draft. CX: Project administration, Supervision, Writing – review & editing.
